# Thoracic Surgery Information on the Internet: A Multilingual Quality Assessment

**DOI:** 10.2196/ijmr.6732

**Published:** 2017-05-12

**Authors:** Myles Davaris, Stephen Barnett, Robert Abouassaly, Nathan Lawrentschuk

**Affiliations:** ^1^ University of Melbourne Melbourne Australia; ^2^ University Hospitals Case Medical Centre Cleveland, OH United States; ^3^ Austin Hospital Department of Surgery University of Melbourne Melbourne Australia

**Keywords:** thoracic, Internet, multilingualism, language, websites

## Abstract

**Background:**

Previous data suggest that quality of Internet information regarding surgical conditions and their treatments is variable. However, no comprehensive analysis of website quality exists for thoracic surgery.

**Objective:**

The aim of this study was to quantify website quality in a multilingual setting using an international standard for assessment.

**Methods:**

Health On the Net (HON) principles may be applied to websites using an automated toolbar function. We used the English, French, Spanish, and German Google search engines to identify 12,000 websites using keywords related to thoracic conditions and procedures. The first 150 websites returned by each keyword in each language were examined. We compared website quality to assess for tertile (is the quality better in first, second, or third 50 websites returned) and language differences. A further analysis of the English site types was undertaken performing a comparative analysis of website provider types.

**Results:**

Overall, there are a considerable number of websites devoted to thoracic surgery: “lung cancer” returned over 150 million websites. About 7.85% (940/11,967) of websites are HON-accredited with differences by search term (*P*<.001) and tertiles (*P*<.001) of the first 150 websites, but not between languages. Oncological keywords regarding conditions and procedures were found to return a higher percentage of HON-accreditation. The percentage of HON-accredited sites was similar across all four languages (*P*=.77). In general, the first tertile contained a higher percentage of HON-accredited sites for every keyword.

**Conclusions:**

Clinicians should appreciate the lack of validation of the majority of thoracic websites, with discrepancies in quality and number of websites across conditions and procedures. These differences appear similar regardless of language. An opportunity exists for clinicians to participate in the development of informative, ethical, and reliable health websites on the Internet and direct patients to them.

## Introduction

### Background

As patients are diagnosed with serious conditions and await complex procedures, it is accepted that they inherently will explore the Internet for answers. Over 80% of patients, health care professionals, and other invested groups utilize the Internet to seek medical information, seeing it as a reliable, trustworthy, and accessible source [1-3]. Industry groups, clinicians, and health care institutions may construct websites with commercial interests in mind [1,4]. In contrast, only a minority of websites are sponsored by government or educational organizations and nonprofit organizations, which may provide objective, unbiased, and hence more accurate information, compared with other sponsors [5,6]. Therefore, the Internet’s accessible source of health information, and frequency of use by the majority, substantiates the need to assess its quality and validity.

Thoracic surgery is a common mode of treatment for many patients with lung cancer. However, patients face a range of extensive and unregulated information regarding conditions and procedures on the Internet, often affecting their expectations and informed decision-making [7,8]. Moreover, language affects the quality of information [9-14], which impacts on multicultural societies and non-English speaking patients who require reliable information.

### Health on the Net

Clinicians also require tools both to identify quality information for themselves and also to direct their patients to reliable, high quality Internet resources [11-13]. High quality and reliable health information can be found through the help of several tools [10,11,15]. The Health On the Net (HON) Foundation is one such tool. HON is a not-for-profit multilingual accreditation body that aims to accredit health websites according to its key principles of authority, complementarity, confidentiality, attribution, justifiability, transparency of authorship, sponsorship, and advertising [11]. The HONcode offers directions for users in evaluating and creating a trustworthy and reputable website [16,17]. Of note, website quality has been tested using the HONcode tool across a range of specialties with only a small percentage of websites (7-27%) being routinely accredited [6,18-20].

A comprehensive literature search regarding website information within the sphere of thoracic surgery was undertaken, yielding no studies that evaluate the quality of thoracic surgery-related information on the Internet. In this study, we aimed to evaluate the quality of current Internet information on thoracic surgery websites based on HON principles, and to compare differences between English, French, German, and Spanish language sites. The effect of language relates to what websites appear on different Google search engines (English, French, German, and Spanish), and whether there are any differences in HON-accredited websites. Our secondary goal was to assess and compare information quality based on types of website sponsors.

## Methods

### Search Engine and Search Terms

Our methodology has been previously described and used [11-13,21]. On this occasion, however, we used the corresponding Google search engine for each respective language search. We performed an Internet search of 20 terms in December 2014 to March 2015 (Table 1) and assessed 12,000 websites. As formal medical terminology has been used for search terms, the same search term used in English was used for the French, German, and Spanish searches on their respective Google search engines. The terms searched were “pectus excavatum,” “pectus carinatum,” “Nuss procedure,” “Ravitch procedure,” “Lorenz bar repair,” “lung cancer,” “nonsmall cell lung cancer,” “small cell lung cancer,” “VATS,” “video-assisted thoracic surgery,” “lung resection,” “lung wedge resection,” “pneumonectomy,” “thoracotomy,” “mediastinoscopy,” “bronchoscopy,” “EBUS,” “endobronchial ultrasound,” and “lung lobectomy.” An expert thoracic surgeon deemed these terms the most common and pertinent medical conditions and procedures for review in this study. These search terms were selected because they are the most objective terms that patients would hear during a consultation. By searching these terms, more meaningful data from websites can be ascertained. Ethics or Institutional Review Board (IRB) approval was not required for this study, since it does not involve patients but only Web-based review of publicly accessible websites.

### Internet Searching for Accredited Websites

International and independent, qualified accrediting bodies check HON status at regular times, ensuring that HON certification meets the strict internationally accepted requirements. Moreover, the HON function has been evaluated by many studies, and judged to be a high caliber tool [10-13,22,23].

Access beyond the first page of results by patients is rare [24]. Thus, the first 150 websites yielded by each search were identified and sequentially screened for quality as defined by the HON Foundation. HON principles through the HONcode toolbar function (downloaded from http://www.hon.ch/ for use on any personal computer. HONcode toolbar is easily installed, providing an accessible and visual cue for users) were then applied. According to the HON Foundation website [10], there are 8 criteria evaluated for HONcode certification of a website. These are (1) authoritative (indicate qualifications of authors), (2) complementarity (information should support, not replace, the doctor-patient relationship), (3) privacy (respect privacy and confidentiality of personal data submitted to the site by visitor), (4) attribution (cite the sources of published information, date medical and health pages), (5) justifiability (site must back up claims relating to benefits and performance), (6) transparency (accessible presentation, accurate email contact), (7) financial disclosure (identify funding sources), and (8) advertising policy (clearly distinguish advertising from editorial content). This toolbar automatically activates if a website is accredited by the HON Foundation (HONcode+), as opposed to the toolbar not lighting up, indicating that the website is not HON-accredited (HONcode−). On the basis of the previous studies, approximately 5% of websites could be deemed HONcode+, but have not been accredited yet [10-13].

### Analysis of Accredited Websites’ Likelihood of Being Viewed

A secondary analysis of the first 150 websites encountered for each search term was undertaken, as previously described [6,18,25]. First, all returned websites for each search term were divided into tertiles (first 50, middle 50, and last 50). The proportion of accredited sites in each tertile and language was then analyzed and compared by the chi-square test. The purpose of this analysis was to determine whether accredited websites were appearing preferentially—that is, in the pages least likely (last 50) versus most likely (first 50) to be viewed.

### Quality Control

For quality control, an English-language search of the control term, “lung cancer,” had nonaccredited sites within the first 150 discovered websites manually evaluated using the HON criteria to determine their HON status to ascertain if they fulfilled the criteria despite not being officially accredited.

### Logistic Regression Examining Variables Associated With HON Status

This test was conducted using the three major variables of our study, namely a search term, language, and tertile, of the first 150 websites returned. The reference groups for each variable were excavatum, the first tertile, and English, respectively.

### Analysis of Website Sponsors

For all search terms, an analysis was undertaken from English-language websites to determine who the website sponsors were. Only English language websites were examined due to the authors’ lack of proficiency in the other languages. The site sponsors were organized into the following groups: (1) lawyers, (2) nonprofit organizations, (3) government organizations or educational institutions, (4) commercial, (5) thoracic specialists and their professional organizations, (6) Books, articles, and references, (7) other health care professionals, (8) other (social media, forums, personal websites, newspapers, and (9) unrelated.

Sponsorship was determined independently by information on the retrieved Web page regarding its origin; if sponsorship was not obviously apparent, the website was explored until sponsorship could be determined. The concept of sponsorship is not to be confused with the Google terminology of “sponsored links,” which either highlights pages at the start of retrieved search or lists links on the side of the page under a banner. As in a previous analysis, such pages were not included in this study [11].

### Statistical Analysis

Comparisons of proportions across types of cancer and language were performed by the chi-square test (or Fisher exact test when counts were <5). All statistical tests were two-sided. Odds ratio and 95% CI were also calculated from the logistic regression analysis. The data analysis for this study was generated by SAS software version 9.1. (SAS Institute Inc).

## Results

### Internet Search Results for Accredited Websites

The total number of websites for each thoracic surgery-related search term is variable (Table 1). “Lung cancer” had the most websites with approximately 150 million websites followed by “small cell lung cancer” with approximately 112 million websites. “Ravitch procedure” returned the least number, with only 159,890 websites.

The total percentage of HON-accredited sites was notably low across all search terms (median 8%; see Table 1). “Lorenz bar repair,” “EBUS,” “endobronchial ultrasound,” and “VATS” had less than 5% of HON-accredited sites (Table 1).

**Figure 1:**
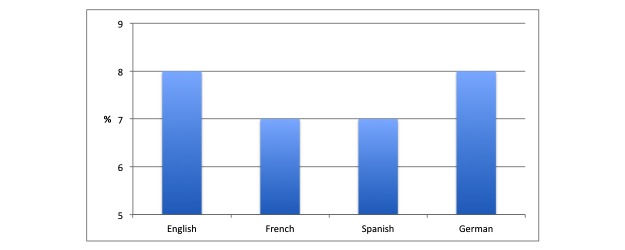
Column graph of median percentage of Health On the Net (HON)–accredited sites for all keywords arranged according to language. Each keyword was searched on native Google search engine of respective countries. The graph indicates the median percentage of HON-accredited websites.

Regarding linguistic differences (see Table 2 and Figure 1), there was a similar number of HON-accredited thoracic websites across all languages evaluated. English (8%) and German (8%), French (7%) and Spanish (7%) had a similar percentage of HON-accredited sites.

**Figure 2:**
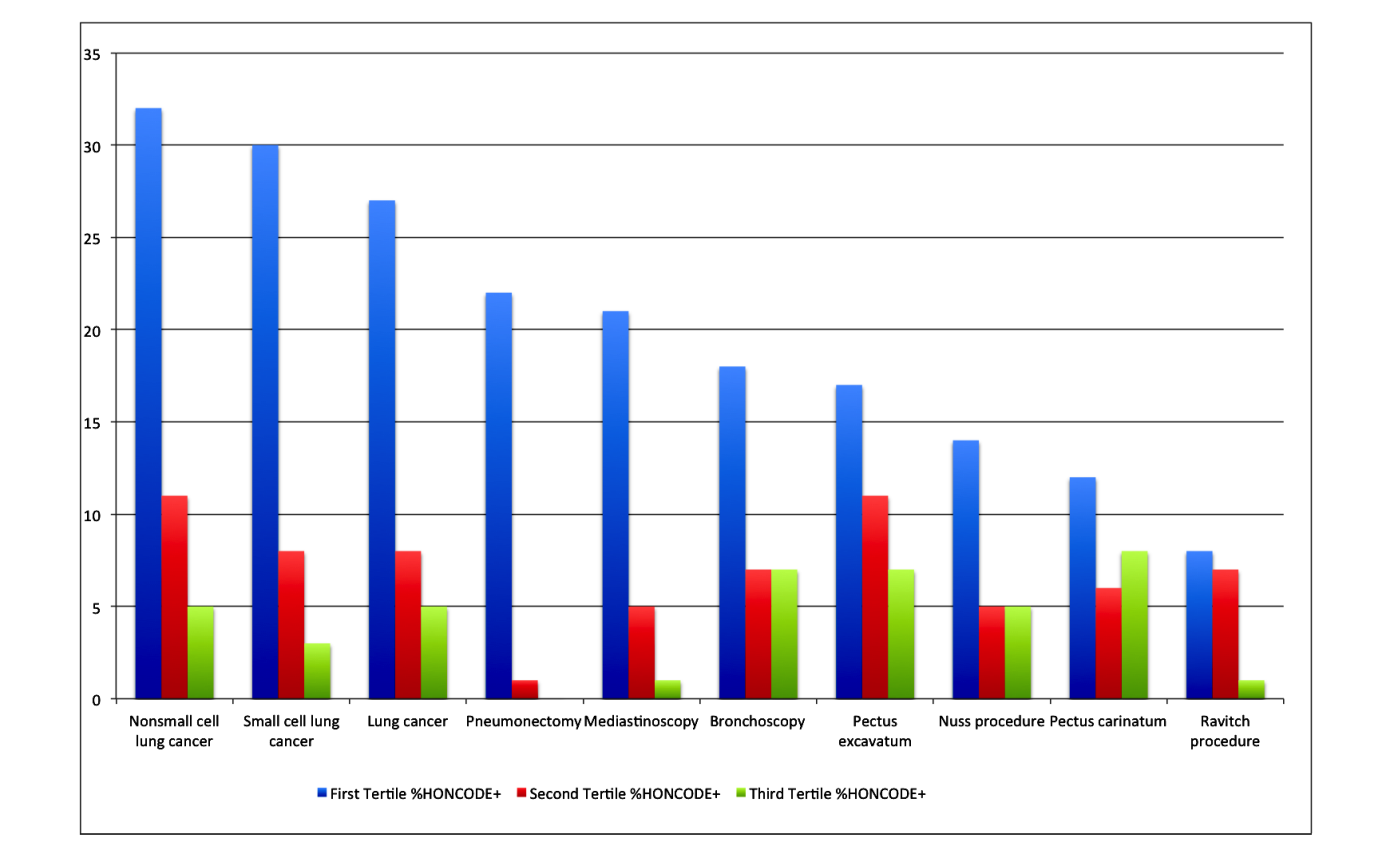
Clustered column graph of percentage of Health On the Net (HON)–accredited websites for keywords arranged by tertiles. The color “blue” indicates percentage HON-accredited websites in first tertile, “red” indicates percentage HON-accredited websites in second tertile, and “green” indicates percentage HON-accredited websites in third tertile.

Tertiles were examined to ascertain where HON-accredited websites were more likely to appear. HON accreditation was seen statistically more commonly in the first tertile (0-50 sites) of websites (see Table 3 and Figure 2).

**Table 1:** Number and percentage of HON-accredited websites.

Category	Search term	Total websites returned	HON^a^-accredited (600 per term)		Total	HONcode%^d^	*P* value
			HONcode+^b^	HONcode−^c^			
**Anatomy**							
	Pectus carinatum	1,069,000	49	551	600	8	
	Pectus excavatum	2,120,000	68	532	600	11	
	Total	1,594,500^e^	117^f^	1083^f^	1200^f^	10^e^	.06
**Approach**							
	Thoracotomy	2,596,000	46	554	600	8	
	Total	2,596,000^e^	46^f^	554^f^	600^f^	8^e^	.55
**Cancer**							
	Lung cancer	149,500,000	79	521	600	13	
	Nonsmall cell lung cancer	67,600,000	96	504	600	16	
	Small cell lung cancer	111,500,000	80	520	600	13	
	Total	111,500,000^e^	255^f^	1545^f^	1800^f^	13^e^	.29
**Endoscopy**							
	VATS	26,320,000	26	574	600	4	
	Video-assisted thoracic surgery	1,934,000	40	560	600	7	
	Total	14,127,000^e^	66^f^	1134^f^	1200^f^	6^e^	.08
**Imaging**							
	EBUS	2,293,000	15	585	600	3	
	Endobronchial ultrasound	793,000	24	576	600	4	
	Total	1,543,000^e^	39^f^	1161^f^	1200^f^	4^e^	.29
**Lungsurg**							
	Lung lobectomy	1,840,000	48	552	600	8	
	Lung resection	22,310,000	32	568	600	5	
	Lung wedge resection	928,000	44	556	600	7	
	Pneumonectomy	3,889,000	44	523	567	8	
	Total	2,864,500^e^	168^f^	2199^f^	2367^f^	8^e^	.26
**Surganatomy**							
	Lorenz bar repair	1,529,000	12	588	600	2	
	Nuss procedure	512,200	47	553	600	8	
	Ravitch procedure	241,400	30	570	600	5	
	Total	512,200^e^	89^f^	1711^f^	1800^f^	5^e^	<.001
**Scope**							
	Bronchoscopy	9,204,000	62	538	600	10	
	Mediastinoscopy	764,000	52	548	600	9	
	Thoracoscopy	1,576,000	46	554	600	8	
	Total	1,576,000^e^	160^f^	1640^f^	1800^f^	9^e^	.26
**Grand total**		2,027,000^e^	940^f^	11027^f^	11967^f^	8^e^(2-16)	<.001

^a^HON: Health On the Net.

^b^HONcode+: HON-accredited website.

^c^HONcode−: not HON-accredited website.

^d^HONcode%: percentage of HON-accredited websites, calculated by ([HONcode+]/[total websites]); where, total websites=(HONcode+)+(HONcode−).

^e^Median.

^f^Sum.

**Table 2:** Percentage of HON-accredited websites by language.

Category	Search terms	English			French				German				Spanish					*P* value	
		+^a^	−^b^	%^c^		+	−	%		+	−	%		+	−	%			
**Anatomy**																			
	Pectus carinatum	13	137	9		14	136	9		12	138	8		10	140	7			.80
	Pectus excavatum	17	133	11		19	131	13		16	140	7		16	134	11			
	Total	30^e^	270^e^	10^d^		33^e^	267^e^	11^d^		28^e^	278^e^	8^d^		26^e^	274^e^	9^d^			
**Approach**																			
	Thoracotomy	14	136	9		10	140	7		11	139	7		11	139	7			
	Total	14^e^	136^e^	9^d^		10^e^	140^e^	7^d^		11^e^	139^e^	7^d^		11^e^	139^e^	7^d^			.84
**Cancer**																			
	Lung cancer	20	130	13		19	131	13		20	130	13		20	130	13			
	Nonsmall cell lung cancer	32	118	21		24	126	16		18	132	12		22	128	15			
	Small cell lung cancer	27	123	18		19	131	13		18	132	12		16	134	11			
	Total	79^e^	371^e^	18^d^		62^e^	388^e^	13^d^		56^e^	394^e^	12^d^		58^e^	392^e^	13^d^			.11
**Endoscopy**																			
	VATS	4	146	2		7	143	5		7	143	5		7	143	5			
	Video-assisted thoracic surgery	11	139	7		10	140	7		10	140	7		10	140	7			
	Total	15^e^	285^e^	5^d^		17^e^	283^e^	6^d^		17^e^	283^e^	6^d^		17^e^	283^e^	6^d^			.98
**Imaging**																			
	EBUS	5	145	3		3	147	2		2	148	1		5	145	3			
	Endobronchial ultrasound	6	144	4		6	144	4		6	144	4		6	144	4			
	Total	11^e^	289^e^	4^d^		9^e^	291^e^	3^d^		8^e^	292^e^	5^d^		11^e^	289^e^	4^d^			.87
**Lungsurg**																			
	Lung lobectomy	11	139	7		13	137	9		11	139	7		13	137	9			
	Lung resection	9	141	6		9	141	6		6	144	4		8	142	5			
	Lung wedge resection	9	141	6		11	139	7		11	139	7		13	137	9			
	Pneumonectomy	13	137	9		10	122	7		12	123	8		9	141	6			
	Total	42^e^	558^e^	7^d^		43^e^	539^e^	7^d^		40^e^	545^e^	7^d^		43^e^	557^e^	8^d^			.99
**Surganatomy**																			
	Lorenz bar repair	3	147	2		3	147	2		3	147	2		3	147	2			
	Nuss procedure	9	141	6		13	137	9		14	136	9		11	139	7			
	Ravitch procedure	6	144	4		9	141	6		8	142	5		7	143	5			
	Total	18^e^	432^e^	4^d^		25^e^	425^e^	6^d^		25^e^	425^e^	5^d^		21^e^	429^e^	5^d^			.65
**Scope**																			
	Bronchoscopy	18	132	12		15	135	10		13	137	9		16	134	11			
	Mediastinoscopy	14	136	9		12	138	8		13	137	9		13	137	9			
	Thoracoscopy	12	138	8		11	139	7		12	138	8		11	139	7			
	Total	44^e^	406^e^	9^d^		38^e^	412^e^	8^d^		38^e^	412^e^	9^d^		40^e^	410^e^	9^d^			.88
**Grand total**		253^e^	2747^e^	8^d^		237^e^	2745^e^	7^d^		223^e^	2762^e^	8^d^		227^e^	2773^e^	7^d^			.76

^a^+: HON-accredited website.

^b^−: not HON-accredited website.

^c^%: percentage of HON-accredited websites, calculated by ([HONcode+]/[total websites]), where, total websites=(HONcode+)+(HONcode−).

^d^Median.

^e^Sum.

**Table 3:** Percentage of HON-accredited websites by tertile.

Category	Search term	HON^a^-accredited									*P* value	
		Tertile 1 (sites 1-50)			Tertile 2 (sites 51-100)			Tertile 3 (sites 101-150)				
		+^b^	−^c^	%^d^	+	−	%	+	−	%		
**Anatomy**												
	Pectus carinatum	23	177	12	11	189	6	15	185	8		.08
	Pectus excavatum	33	167	17	22	178	11	13	187	7		<.001
**Approach**												
	Thoracotomy	28	172	14	18	182	9	0	200	0		<.001
**Cancer**												
	Lung cancer	54	146	27	16	184	8	9	191	5		<.001
	Nonsmall cell lung cancer	64	136	32	22	178	11	10	190	5		<.001
	Small cell lung cancer	59	141	30	16	184	8	5	195	3		<.001
**Endoscopy**												
	VATS	12	188	6	5	195	3	9	191	5		.23
	Video-assisted thoracic surgery	30	170	15	6	194	3	4	196	2		<.001
**Imaging**												
	EBUS	3	197	2	4	196	2	8	192	4		.24
	Endobronchial ultrasound	19	181	10	4	196	2	1	199	1		<.001
**Lungsurg**												
	Lung lobectomy	31	169	16	4	196	2	13	187	7		<.001
	Lung resection	20	180	10	9	191	5	3	197	2		<.001
	Lung wedge resection	21	179	11	16	184	8	7	193	4		.02
	Pneumonectomy	43	157	22	1	199	1	0	167	0		<.001
**Surganatomy**												
	Lorenz bar repair	12	188	6	0	200	0	0	200	0		<.001
	Nuss procedure	28	172	14	9	191	5	10	190	5		<.001
	Ravitch procedure	15	185	8	13	187	7	2	198	1		<.001
**Scope**												
	Bronchoscopy	35	165	18	13	187	7	14	186	7		<.001
	Mediastinoscopy	41	159	21	9	191	5	2	198	1		<.001
	Thoracoscopy	37	163	19	7	193	4	2	198	1		<.001
**Grand total**		608^f^	3392^f^	15^e^	205^f^	3795^f^	5^e^	127^f^	3840^f^	3^e^		<.001

^a^HON: Health On the Net.

^b^+: HON-accredited website.

^c^−: not HON-accredited website.

^d^(%): percentage of HON-accredited websites.

^e^Median.

^f^Sum.

#### Quality Control

For the first 150 “lung cancer” (English) results, we found that 20 sites were accredited by the HON toolbar and 130 were not. We found that 6.9% (9/130) of those nonaccredited sites met HON criteria when assessed manually and 13.2% (79/600) of cancer-related thoracic surgery websites are HON-accredited.

#### Logistic Regression Examining Variables Associated With HON Status

Odds ratios calculated by search term, language, tertile, and between groups, highlighted significant differences (Table 4). For language, English compared with French, German, or Spanish was just as likely to return an accredited site. The second tertile of websites (51-100) assessed were more likely than the third tertile (101-150) to have accredited sites.

**Table 4:** Odds ratio and 95% CI. Illustration of odds ratio of a search having HON-accreditation in relation to referent. The higher the ratio, the less likely a search term has HON-accreditation. The lower the ratio, the more likely a search term has HON-accreditation.

Effect on HONcode status		Odds ratio	95% CI
**Search terms**			
	Excavatum	1.00 (referent)	
	Bronchoscopy	1.116	0.767-1.622
	EBUS	5.239	2.940-9.334
	Endobronchial ultrasound	3.197	1.964-5.207
	Lorenz bar repair	6.598	3.512-12.394
	Lung cancer	0.834	0.584-1.191
	Lung lobectomy	1.498	1.007-2.229
	Lung resection	2.347	1.505-3.662
	Lung wedge resection	1.652	1.100-2.481
	Mediastinoscopy	1.368	0.926-2.020
	Nonsmall cell lung cancer	0.653	0.463-0.922
	Nuss procedure	1.534	1.029-2.288
	Pneumonectomy	1.612	1.073-2.422
	Ravitch procedure	2.517	1.599-3.962
	Small cell lung cancer	0.821	0.576-1.172
	Thoracoscopy	1.572	1.052-2.349
	Thoracotomy	1.572	1.052-2.349
	VATS	2.936	1.826-4.720
	Video-assisted thoracic surgery	1.838	1.211-2.788
	Carinatum	1.464	0.985-3.960
**Websites^a^**			
	First tertile (0-50)	1.00 (referent)	
	Second tertile (51-100)	3.354	2.840-3.960
	Third tertile (101-150)	5.522	4.531-6.730
**Language**			
	English	1.00 (referent)	
	French	1.076	0.889-1.303
	German	1.155	0.951-1.402
	Spanish	1.134	0.935-1.375

^a^Sum.

**Table 5:** Website sponsor analysis.

Search term	Lawyer, (%)	Non-profit, (%)	Government or education, (%)	Commercial, (%)	Thoracic specialists or professional organizations, (%)	Books, articles, references, (%)	Other health care professionals, (%)	Others (social media, forums, personal websites, newspapers), (%)	Unrelated, (%)	*P* value
Carinatum	0 (0)	13 (9)	48 (32)	17 (11)	10 (7)	51 (34)	3 (2)	8 (5)	0 (0)	.35
Excavatum	0 (0)	12 (8)	61 (41)	11 (7)	7 (5)	53 (35)	0 (0)	6 (4)	0 (0)	
Thoracotomy	0 (0)	7 (5)	38 (25)	4 (3)	6 (4)	83 (55)	0 (0)	12 (8)	0 (0)	N/A
Lung cancer	0 (0)	23 (15)	48 (32)	3 (2)	3 (2)	52 (35)	0 (0)	21 (14)	0 (0)	<.001
Nonsmall cell lung cancer	0 (0)	18 (12)	40 (27)	8 (5)	1 (1)	79 (53)	0 (0)	4 (3)	0 (0)	
Small cell lung cancer	1 (1)	10 (7)	49 (33)	6 (4)	2 (1)	75 (50)	0 (0)	7 (5)	0 (0)	
VATS	0 (0)	4 (3)	38 (25)	5 (3)	6 (4)	23 (15)	0 (0)	2 (1)	72 (48)	.001
Video-assisted thoracic surgery	0 (0)	2 (1)	79 (53)	4 (3)	7 (5)	58 (39)	0 (0)	2 (1)	0 (0)	
EBUS	0 (0)	1 (1)	30 (20)	11 (7)	5 (3)	34 (23)	0 (0)	1 (1)	68 (45)	<.001
Endobronchial ultrasound	0 (0)	2 (1)	53 (35)	6 (4)	6 (4)	76 (51)	0 (0)	7 (5)	0 (0)	
Lung lobectomy	1 (1)	9 (6)	44 (29)	4 (3)	5 (3)	70 (47)	0 (0)	17 (11)	0 (0)	.001
Lung resection	0 (0)	4 (3)	35 (23)	2 (1)	5 (3)	101 (67)	0 (0)	3 (2)	0 (0)	
Lung wedge resection	0 (0)	14 (9)	33 (22)	2 (1)	5 (3)	86 (57)	0 (0)	10 (7)	0 (0)	
Pneumonectomy	1 (1)	6 (4)	17 (11)	2 (1)	4 (3)	109 (73)	0 (0)	11 (7)	0 (0)	
Lorenz bar repair	4 (3)	3 (2)	13 (9)	5 (3)	2 (1)	72 (48)	0 (0)	3 (2)	48 (32)	<.001
Nuss procedure	0 (0)	5 (3)	29 (19)	2 (1)	5 (3)	84 (56)	0 (0)	25 (17)	0 (0)	
Ravitch procedure	1 (1)	4 (3)	40 (27)	9 (6)	3 (2)	66 (44)	0 (0)	27 (18)	0 (0)	
Bronchoscopy	0 (0)	4 (3)	58 (39)	12 (8)	9 (6)	62 (41)	0 (0)	5 (3)	0 (0)	<.001
Mediastinoscopy	0 (0)	6 (4)	38 (25)	6 (4)	4 (3)	87 (58)	0 (0))	9 (6)	0 (0)	
Thoracoscopy	1 (1)	2 (1)	30 (20)	15 (10)	5 (3)	78 (52)	0 (0)	19 (13)	0 (0)	
Total mean (mean %)	9 (<1)	149 (5)	821 (27)	134 (4)	100 (3)	1399 (47)	3 (<1)	199 (7)	188 (6)	<.001

#### Analysis of Website Sponsors

The sponsor analysis of the 150 websites in English (Table 5) indicated that the most commonly encountered sponsors were “books, articles, and references” (47.1%, 1399/2967) followed by “government or education” (27.7%, 821/2967), “others (social media, forums, personal websites, newspapers” (6.7%, 199/2967), “nonprofit organizations” (5.0%, 149/2967), “commercial” (4.5%, 134/2967), and “thoracic specialists or professional organizations” (3.4%, 100/2967). “Lawyer” (<1%, 9/2967) and “other health care professionals” (<1%, 3/2967) sponsored far less sites. A small percentage (6.3%, 188/2967) of sponsor websites were unrelated to medicine.

Search terms with a larger percentage of “government or education” or “books, articles, and references” were the terms with a larger percentage of HON-accredited websites: “lung cancer,” “nonsmall cell lung cancer,” “small cell lung cancer” with *P* value <.001; “lung lobectomy,” “lung resection,” and “lung wedge resection” with *P* value .001; “pneumonectomy,” “bronchoscopy,” and “thoracoscopy” with *P* value .001.

## Discussion

### Principal Findings

The aim of this study was to quantify information quality on thoracic surgery-related websites on the Internet. Clinicians may become aware of the lack of quality information regarding thoracic surgery and help to educate patients about the pitfalls of information on the Internet, and direct them to better quality websites.

In summary, the total number of websites for keyword searches varies considerably. The total percentage of HON-accredited websites was markedly low across all search terms. There were minimal linguistic differences in HON-accredited websites, with HON-accredited websites most likely to appear in the first tertile. Nearly half of the websites were books, articles, or references, whereas nearly one-third were governmental or educational.

### Comparison With Prior Work

The Internet has developed into an accessible source of health information for everyone. Health websites are guides for patients wanting to better understand their conditions [26]. Web-based health information was sought by 72% of adult Internet users over the last few years [27], a number predicted to grow. Clinicians directing patients to reliable information has many benefits: improving patient-doctor relationships, reinforcing consultation discussions, assisting informed decision-making, providing education before and after events, and helping patients seek appropriate consultation for sensitive topics (eg, urology, gynecology).

There is a stark discrepancy between reliable health information and quality resources that disseminate it. The number of websites providing accurate information for thoracic surgery is not ideal. Only 13% of cancer-related thoracic surgery websites overall were HON-accredited. This is less than in our previous studies, uro-oncology websites [6] in 2009 and surgical oncology websites in 2012 [18], which each returned 18% of oncology-related HON-accredited websites. Similarly, there were 15% of HON-accredited gynecological oncology-related websites [20]. Worse still, only 9% of benign prostate hyperplasia websites were HON-accredited [19]. This reflects our hypothesis that reliable, high-quality health information on the Internet is lacking, specifically for thoracic surgery as well as in a broader context. In the latter study [19], only 7% of nononcology-related websites such as “surgical treatments” were HON-accredited. This figure is comparable with our 10% “Lungsurg” HON-accredited websites. These results are concerning because they imply that patients will encounter unreliable information about their condition, regardless of cancer type. Evidently, this makes website assessment difficult for patients and clinicians alike, potentially leading to distrust of Internet thoracic surgery resources.

It has previously been acknowledged that website quality differs by language [10,11,14]. In our study, whereas English language searches returned more websites overall, both German and English searches returned 8% HON-accredited sites, and French and Spanish searches returned 7% HON-accredited sites. Thoracic surgery information is far more uniform across languages than results from our previous studies [6,18-20], albeit still alarmingly low. It is evident that there is a paucity of high quality, comprehensive information on thoracic surgery available around the world on the Internet, regardless of language. Similarly, HON-accredited websites are more likely to appear in the first tertile overall than in the second or third tertiles. This tertile discrepancy was expected since the Google algorithm generally places the most relevant websites first. Further analysis into the proportion of HON-accredited websites on the first page compared with the first tertile overall may yield interesting results, since it has been known that patients rarely move past the first search page.

Websites also act as a conduit for advertising. Health information is increasingly being controlled by marketing and commercial interests, taking advantage of a significant proportion of the population searching for health information [28]. Consequently, unbiased views are sacrificed for the type of health information offered. However, the majority of sponsors in this study were composed of (1) academic books, articles, and references and (2) government or education. The absence of commercial bodies or marketing in this area implies that thoracic surgery information might not be biased or skewed for marketing purposes, compared with other medical fields previously analyzed [6,18-20]. Notably, the search terms with these sponsors were those with more website results and more HON-accredited websites. This suggests a conscious effort to provide high quality information for these conditions and procedures. Although our study only revealed 1% of websites sponsored by lawyers, a search performed in the United States may show otherwise. This illustrates the unpredictable nature of the Internet.

HONcode is a simple means by which a clinician or patient can objectively correlate a website with high quality information. Compared with other instruments for evaluating website quality, it appears to be a straightforward, valuable tool, and fulfills its goal of identifying reliable health websites [29]. However, HONcode is by no means the only way to rate quality. The DISCERN instrument [30] and LIDA tool [31] are freely available online, designed to help users evaluate the quality of health information on the Internet. The ODPHP’s National Quality Health Website Survey instrument provides a sophisticated method to assess website quality, though is quite time-consuming and subjective [32]. Thus, compared with other, more intensive search tools, HONcode can be used to access reliable information easily by patients and clinicians, who have no prior experience or knowledge. Furthermore, it has been previously shown that website affiliation with HONcode is a significant predictor for scientific information quality [23]. Due to the growing number of websites, the HONcode certification seal is now obtained by voluntary application. However, many high quality websites lack the HONcode seal. In our study, 6% of websites in the control term could have met the criteria and this is consistent with prior research [6,18-20]. Currently, no studies evaluate awareness of HON certification in organizations and patients. Hence, shortcomings of HON may include voluntary application and lack of public awareness. Patients may bypass trustworthy websites, whereas organizations may not actively apply for HON certification. In a wider context, there is a notable lack of congruence of criteria between health information quality assessment tools [33]. Future research may be needed to streamline assessment tools, or streamline health website guidelines so that quality information is standardized. However, this is out of the scope of this paper. More immediately, further research is required to anal awareness of HON. Depending on these results, appropriate steps could then be taken to help clinicians, patients, and organizations to be exposed to HONcode, enabling access to reliable sources of information.

### Limitations

It must be said that HONcode is a predictive indictor for high quality websites, which has its drawbacks. Thus, a proportion of websites with objectively high quality information may not fulfill HONcode criteria, and vice versa. As of 2015, HONcode certification is provided as a paid service. This can distort the validity of website information with HONcode criteria.

An inherent limitation of this study involves the search terms used. It cannot be guaranteed that patients would use these terms in their own research of their condition. It is in dispute whether informal search terms would yield websites with better quality information. Conversely, it may result in unrelated website results. However, given that the search terms used in this study are the most formal and objective, informal search terms would likely defer to pages with the formal terms by the Google search algorithm. One solution to this limitation is to encourage clinicians to use the formal medical terms for their patients, thereby empowering patients to research their condition better, ultimately resulting in greater patient education.

As with any Internet study, its dynamic and diverse character produces inherent limitations. In our study, we only performed searches in Melbourne, Australia. It would be interesting to perform multiple searches at various times and locations, analyzing any differences found. “Google” is the most popular search engine (http://searchenginewatch.com), having been used in other studies [10]. However, studies have also shown the impact of social media and health-related videos on YouTube on health care [34]. As these media are not appropriately standardized for health promotion and education, these studies highlight the need for caution among users. Search engines rely on language filters to determine sites returned, but Google enables a multilingual approach. A key advantage of Google may be for clinicians and patients who speak the languages analyzed here, which have a low number of accredited websites. Google translate may provide people with wider access to information online, though quality may vary. The impact of the validity of HON certification once a website has been translated by Google was not investigated in this study.

### Conclusions

In conclusion, clinicians must appreciate the lack of validated information of most thoracic surgery websites. Discrepancies are apparent in quality and number of websites between search terms, tertiles, and language. Awareness of this lack of quality can facilitate clinicians in educating patients by using the formal medical term to empower patients to research their condition more comprehensively and thus gain a greater level of understanding. Clinicians must be proactive in identifying and directing patients to trustworthy and accurate information on websites. HONcode is an uncomplicated search tool and can serve as the vanguard to detect appropriate and trustworthy websites.

## Abbreviations

HON: Health On the Net
HONcode: toolbar function for website accreditation recognition by HON principles
IRB: Institutional Review Board
PoHONA: percentage of HON-accredited sites
WHO: World Health Organization
